# Intracellular Delivery of Natural Antioxidants via Hyaluronan Nanohydrogels

**DOI:** 10.3390/pharmaceutics11100532

**Published:** 2019-10-14

**Authors:** Elita Montanari, Chiara Di Meo, Tommasina Coviello, Virginie Gueguen, Graciela Pavon-Djavid, Pietro Matricardi

**Affiliations:** 1 Department of Drug Chemistry and Technologies, Sapienza University of Rome, P.le Aldo Moro 5, 00185 Rome, Italy; elita.montanari@uniroma1.it (E.M.); tommasina.coviello@uniroma1.it (T.C.); pietro.matricardi@uniroma1.it (P.M.); 2 INSERM U1148, Laboratory for Vascular Translational Science, Cardiovascular Bioengineering, Paris 13 University, Sorbonne Paris Cite 99, Av. Jean-Baptiste Clément, 93430 Villetaneuse, France; virginie.gueguen@univ-paris13.fr

**Keywords:** drug delivery, astaxanthin, resveratrol, curcumin, hyaluronan, nanohydrogels, oxidative stress, intracellular therapy

## Abstract

Natural antioxidants, such as astaxanthin (AX), resveratrol (RV) and curcumin (CU), are bioactive molecules that show a number of therapeutic effects. However, their applications are remarkably limited by their poor water solubility, physico-chemical instability and low bioavailability. In the present work, it is shown that self-assembled hyaluronan (HA)-based nanohydrogels (NHs) are taken up by endothelial cells (Human Umbilical Vein Endothelial Cells, HUVECs), preferentially accumulating in the perinuclear area of oxidatively stressed HUVECs, as evidenced by flow cytometry and confocal microscopy analyses. Furthermore, NHs are able to physically entrap and to significantly enhance the apparent water solubility of AX, RV and CU in aqueous media. AX/NHs, RV/NHs and CU/NHs systems showed good hydrodynamic diameters (287, 214 and 267 nm, respectively), suitable ζ-potential values (−45, −43 and −37 mV, respectively) and the capability to neutralise reactive oxygen species (ROS) in tube. AX/NHs system was also able to neutralise ROS *in vitro* and did not show any toxicity against HUVECs. This research suggests that HA-based NHs can represent a kind of nano-carrier suitable for the intracellular delivery of antioxidant agents, for the treatment of oxidative stress in endothelial cells.

## 1. Introduction

Reactive oxygen species (ROS) are highly reactive molecules derived from the molecular oxygen [1], which are produced as normal by-products of aerobic respiration in eukaryotic cells. Although physiological concentrations of ROS are crucial for ensuring the cell survival, the loss of redox equilibrium by over-production of ROS or by failed control systems is detrimental to cells and leads to oxidative stress (OS) [2]. Many research lines evidenced that OS and inflammation processes are key-factors for the development of several diseases, such as neurodegenerative diseases, cardiovascular disorders and cancer [3]. Epidemiological studies suggest that low levels of antioxidants are associated with an increased risk of disease, whilst an increased consumption seems to be protective. Targeting the reestablishment of redox homeostasis by stimulation/blocking of endogenous systems or by administration of exogenous drugs is a strategy followed in several pathologies. In a recent clinical trial, the association of N-acetylcysteine with metformin revealed good perspectives in the treatment of non-alcoholic steatohepatitis [4]. The combination of anti-inflammatory drugs, such as sulphasalazine [5] with natural antioxidants (e.g. curcumin and quercetin) [6,7], showed significant antioxidant properties in animal models.

Among natural antioxidants, carotenoids (e.g., astaxanthin, AX) present interesting biological properties: AX is a potent quencher of oxygen singlets, superoxide anions and hydroxyl radicals [8]. In previous works we showed that natural AX can protect human umbilical vein endothelial cells (HUVECs) under OS [9]. However, AX is a highly unsaturated molecule and easily decomposes when exposed to heat, light and oxygen. In addition, the applications of AX and other widely used antioxidants molecules, such as resveratrol (RV) [10,11] and curcumin (CU) [12,13], are often limited by their low solubility in water, their physico-chemical instability and their low bioavailability. Therefore, the use of novel drug delivery systems for enhancing the apparent water-solubility, stability and release of antioxidant molecules in the site of interest is crucial for the *in vivo* delivery and the development of antioxidant-based therapies. In this respect, nanohydrogels (NHs) [14,15], which are nano three-dimensional networks, are capable to deliver a variety of bioactive molecules at the target site, such as hydrophobic [16] as well as hydrophilic drugs [17], polypeptides [18] and genetic materials [19]. In fact, the porosity of the NHs network provides a reservoir for loading molecular and macromolecular therapeutics as well as protecting them from the environmental degradation. NHs are usually soft, hydrophilic, biocompatible and are able to absorb a high amount of water and easily swell and de-swell in aqueous media [20]. NHs can be prepared from natural [21] and/or synthetic polymers [22] and, based on the type of linkages present in the polymer network, NHs are subdivided into groups based on either physical or chemical cross-linking. Among the natural polymers, hyaluronan (HA) [23] is a linear, non-sulfated glycosaminoglycan, a poly-anionic polysaccharide, which occurs in all living organisms. HA is highly hydrophilic and can interact with a number of molecules and receptors which are involved in cellular signal transductions [24]. The major HA receptor is CD44 [25], which is known to be involved in the binding, endocytosis and metabolism of HA. A considerable number of publications reported that CD44 is up-regulated and plays a crucial role in a variety of inflammatory diseases [26,27]. However, the real functions of CD44 in inflammation processes are rather complex and they are still under investigation.

The present study aims to investigate the capability of HA-based NHs to enter healthy or oxidatively stressed endothelial cells (HUVECs) and to physically encapsulate three different antioxidant molecules, such as AX, RV and CU. AX/NHs, RV/NHs and CU/NHs systems were characterised in terms of size, polydispersity, ζ-potential and loading efficiency, while their antioxidant activity was investigated in tube. Moreover, the cellular antioxidant activity of AX/NHs and its toxicity were studied in oxidatively stressed HUVECs.

## 2. Materials and Methods

### 2.1. Chemicals

Hyaluronan (HA, M*_w_* = 2.2 × 10^5^) was purchased from Contipro (Dolní Dobroucˇ, Czech Republic) and was modified in the tetrabutylammonium salt (HA^−^TBA^+^) by using a Dowex cation exchange resin (Merck, Milan, Italy). Natural curcumin (CU, purity > 97.0% HPLC) and resveratrol (RV, purity > 99.0% HPLC) were purchased from Tokyo Chemical Industry Co., Ltd. (Tokyo, Japan). Natural astaxanthin from Haematococcus pluvialis (AX, purity > 97.0% HPLC), cholesterol (CH), 4-bromobutyric acid, *N*-methyl-2-pyrrolidone (NMP), *N*-(3- dimethylaminopropyl)-*N*′-(ethylcarbodimide hydrochloride) (EDC∙HCl), 4-(dimethylamino) pyridine (DMAP), dimethyl sulfoxide (DMSO, American Chemical Society (ACS) grade reagent ≥ 99.9%), rhodamine B isothiocyanate (Rhod), 2,2′-azino-di-(3-ethylbenzthiazoline sulfonic acid) (ABTS) solution and potassium persulfate (K_2_S_2_O_8_) were purchased from Sigma-Aldrich (Milan, Italy).

Human umbilical vein endothelial cells (HUVECs, ATCC CRL 1730) were purchased from LGC Standards S.a.r.l. (Molsheim, France). Antimicyn A (Streptomyces sp., AM), phalloidin, 4′,6-diamidino-2-phenylindole (DAPI), Triton, paraformaldehyde (PFA), dihydroethidium dye (DHE), *N*-acetyl cysteine (NAC) were purchased from Sigma-Aldrich (Sigma Aldrich Chemie S.a.r.l., L’Isle d’Abeau Chesnes, France). Phosphate buffer saline (PBS), trypsin, Hank’s Balanced Salt Solution (HBSS) and all reagents for cell culture were purchased from Invitrogen, ThermoFisher Scientific (Villebon sur Yvette, France). Culture plates were from Costar (Fisher Scientific SAS, Illkirch Cedex, France). CellTiter 96® AQueous One Solution Cell Proliferation Assay (MTS) was purchased from Promega (Charbonnières-les-Bains, France).

### 2.2. Methods

#### 2.2.1. Synthesis of Empty and Fluorescent NHs

The methods for the synthesis of hyaluronan-cholesterol (HA-CH) derivative and the preparation of empty nanohydrogels formed by the self-assembling of HA-CH molecules (NHs) were extensively described in previous works [18,20] and will be not explained in detail here.

To obtain fluorescent NHs, the Rhod dye was covalently linked to the hydroxyl groups of NHs as previously reported [17].

#### 2.2.2. Cell Culture

Cells were grown to 70–80% confluence, according to each experimental setting. In all experiments, untreated cells (which received PBS) were used and processed in parallel for an appropriate comparison.

##### Healthy HUVECs

HUVECs were cultured in complete low glucose Dulbecco’s Modified Eagle Medium (DMEM, 1X GlutamaxTM-I) supplemented with 10% Foetal Calf Serum and 1% (*v*/*v*) Penicillin-Streptomycin-Amphotericin (PSA) solution, at 37 °C in a humidified atmosphere with 5% CO_2_.

##### Oxidatively Stressed HUVECs

HUVECs were seeded in well plates and incubated for 48 h in complete DMEM. Cells were washed with PBS and, after the addition of fresh complete DMEM, cells were incubated with a suitable volume of 150 µM AM to obtain the final AM concentration of 11 µM, for 1 h at 37 °C in the dark, thus obtaining oxidatively stressed HUVECs.

#### 2.2.3. Cell Binding/Uptake of NHs in HUVECs

The cell binding/uptake kinetics of fluorescent and empty NHs (Rhod-NHs) was studied in healthy or oxidatively stressed HUVECs, with a BD Accuri C6, BD 254 Bioscences (Erembodegem, Belgium) flow cytometer equipped with a 488 nm excitation laser beam and a 585/40 nm bandpass filter (FL2 channel). For each sample 50,000 events were collected. 300,000 cells/well (1.5 mL of cell suspension in complete DMEM) were seeded in a six-well plate and incubated for 48 h. Cell monolayers were then washed with 1 mL PBS, added by 1.5 mL of fresh complete DMEM and incubated with 0.35 mL of Rhod-NHs in PBS, at the final concentration of 0.1 mg mL^−1^, for 3, 6 or 24 h at 37 °C. As negative control, cells received 0.35 mL PBS. Medium was removed, HUVECs were washed three times with PBS, allowed to detach with 0.7 mL trypsin and finally added to 2 mL complete DMEM. Cell suspensions were centrifuged for 5 min at 1200 rpm at 25 °C. Supernatants were removed and pellets were washed with 1 mL HBSS solution and centrifuged again. Pellets were re-suspended in 0.5 mL HBSS and the red fluorescence (due to the presence of Rhod) was detected with the flow cytometer. For the study of the binding/uptake kinetics in oxidatively stressed HUVECs, prior to the incubation with 0.35 mL, Rhod-NHs cells were added by 0.11 mL of AM solution (150 µM), for 1 h at 37 °C in the dark (AM final concentration = 11 µM), washed with 1 mL PBS and added by 1.5 mL of fresh complete DMEM. For the negative control, HUVECs received 0.35 mL PBS. Results were expressed as median fluorescence intensity (MFI). The dot plot was reported by plotting forward scattering (FSC-H) versus side scattering (SSC-H) and the gate was defined excluding cell debris. Each experiment was performed in triplicate (*n* = 3).

#### 2.2.4. Cell Imaging: Confocal Microscopy

Immunofluorescence signal of Rhod-NHs was analysed by recording stained images using the Carl Zeiss® LSM 780, objective ×10, confocal microscope. Digital images were processed with ZEN software or Fiji-win 32 (ImageJ). 20,000 cells/well (400 µL of cell suspension in complete DMEM) were seeded in a Lab-Tek (Thermo Fisher Scientific, Milan, Italy) and incubated for 48 h. Cell monolayers were then washed with 400 µL PBS, added by 400 µL of fresh complete DMEM and incubated with 29 µL of PBS (healthy HUVECs) or 29 µL of 150 µM AM (oxidatively stressed HUVECs) for 1 h at 37 °C in the dark. Cells were washed with 400 µL PBS, added by 400 µL of fresh complete DMEM and incubated with 100 μL of 500 µg mL^−1^ Rhod-NHs in PBS (corresponding to a final concentration of 100 μg mL^−1^) for 3 h and 24 h. As negative control, cells received 100 μL PBS. HUVECs were then washed with 400 µL PBS, fixed with 200 µL 2% PFA for 10 min at 4 °C, washed twice with 400 µL PBS and added by 200 µL DAPI (dilution 1:50,000) for 5 min at 25 °C. Cells were finally washed twice with 400 µL PBS and slides were mounted with 10 µL Dako and checked with the confocal microscope.

#### 2.2.5. Preparation and Characterisation of AX-Loaded NHs

For the preparation of AX-loaded NHs samples of HA-CH (1.5 mg mL^−1^, degree of functionalisation (Df) = 15%, mols of CH/ mols of HA repeating units) were left under magnetic stirring in bi-distilled water overnight, at 25 °C. The suspensions were then placed in an autoclave (121 °C, 1.1 bar for 20 min) where NHs were formed. AX was solubilised in DMSO (concentration = 9 mg mL^−1^) and then added to NHs suspension (24 µL for 1 mg of HA-CH), corresponding to a weight ratio of 0.216 (mg of AX/mg of NHs). The nano-suspensions of AX/NHs were left for 5 h at 25 °C in the dark, dialysed against water for 6 h (MW cut-off: 12,000–14,000) and then centrifuged at 4000 rpm for 20 min at 25 °C. Pellets (unloaded AX) were discarded and the supernatants (AX/NHs) were freeze-dried. Mean hydrodynamic diameter (d_h_), polydispersity index (PDI) and ζ-potential (ζ-pot) of AX/NHs were measured by Dynamic Light Scattering (DLS) at 25 °C by using a Zetasizer Nano ZS instrument (Model ZEN3690, Malvern Instruments) equipped with a solid state HeNe laser (λ = 633 nm) at a scattering angle of 173°. The electrophoretic mobility of the samples was converted in ζ-pot by using the Smoluchowski equation. Empty NHs were also tested for an appropriate comparison. Each experiment was performed in triplicate (*n* = 3).

#### 2.2.6. Preparation and Characterisation of RV or CU-Loaded NHs

Samples of HA-CH (1.0 mg mL^−1^, Df of 15%) were left under magnetic stirring in bi-distilled water overnight, at 25 °C. The suspensions were then placed in an autoclave (121 °C, 1.1 bar for 20 min) where NHs were formed. RV or CU were solubilised in acetone at the concentration of 2 mg mL^−1^; 0.5 mL of each drug solution were allowed to evaporate with Heidolph Hei-VAP rotary evaporator (Buchi, Schwabach, Germany) and the drug film was then added by 3 mL of NHs, corresponding to a weight ratio of 0.33 (mg of RV or CU / mg of NHs). The mixtures were kept under magnetic stirring for 14 h at 25 °C in the dark and then centrifuged at 4000 rpm for 10 min at 20 °C. Pellets (unloaded RV or CU) were discarded and the supernatants (RV/NHs or CU/NHs) were analysed with DLS. Each experiment was performed in triplicate (*n* = 3).

#### 2.2.7. Quantification of Entrapped AX into NHs

The amount of AX entrapped into NHs was estimated using a Perkin-Elmer double beam “Lambda 3A” model Ultraviolet-Visible (UV-Vis) spectrometer (Milan, Italy). Freeze-dried AX/NHs were previously dispersed in few µL of water and then solubilised in a large excess of DMSO, by vortexing for few minutes. Samples were analysed at 25 °C, using 1 mm quartz cuvettes (Hellma Analytics, Milan, Italy). The AX calibration curve was evaluated at the concentration range of 6.25–100 μg mL^−1^ (R^2^ = 0.999, λ = 490 nm, *n* = 5). Each experiment was performed in triplicate (*n* = 3).

#### 2.2.8. Quantification of Entrapped RV or CU into NHs

RV or CU pellets (free drugs) were solubilised in EtOH and quantified in order to obtain, by difference, the amount of entrapped drug into NHs. Analyses were performed by HPLC using a KnauerAzura instrument equipped with a binary pump (Azura P 6.1L) and a UV-Vis detector (190–750 nm, Azura UVD 2.1L), controlled by Clarity software. Samples (20 μL) were injected into a KnauerEurospher II C18 column (5 μm, 4.6 × 250 mm); the samples were injected at 1 mL min^−1^ in mixtures of water: acetonitrile (gradient mode) from 70:30 to 0:100 for RV and in water (+ 0.1% *v*/*v* of TFA): acetonitrile (+ 0.1% *v*/*v* of TFA) from 50:50 to 0:100 for CU. The unloaded RV was quantified at λ = 306 nm using a calibration curve previously recorded with RV standard solutions in ethanol in the range 6.25–50 μg mL^−1^ (R^2^ = 0.999, *n* = 5); CU was detected at λ = 425 nm using a calibration curve in ethanol (0.39–50 μg mL^−1^, R^2^ = 0.999, *n* = 8) by integrating the three signals related to CU (~79 %), demethoxycurcumin (~18%) and bisdemethoxycurcumin (~3%).

Encapsulation Efficiency (EE) and Drug Loading (DL) of AX/NHs, RV/NHs and CU/NHs were calculated using the Equations (1) and (2):

(1) $$\%EE=\;\frac{concentration\;of\;loaded\;drug}{concentration\;of\;added\;drug}\times \;100$$

(2) $$\%DL=\;\frac{concentration\;of\;loaded\;drug}{polymer\;concentration}\times \;100$$

#### 2.2.9. Antioxidant Activity of AX/NHs, RV/NHs and CU/NHs in Tube

ABTS assay: 5 mL of 3.8 mg mL^−1^ ABTS solution in bi-distilled water were added to 88 µL of 38 mg mL^−1^ K_2_S_2_O_8_ solution in bi-distilled water, corresponding to a molar ratio of 2.3 (mol of ABTS per mol of K_2_S_2_O_4__8_). The mixture (containing ABTS^•^^+^) was left overnight at 25 °C in the dark and diluted ~1:80 with bi-distilled water. 2.9 mL of the diluted ABTS^•^^+^ solution were added by 0.1 mL of nano-suspensions in bi-distilled water (AX/NHs, RV/NHs or CU/NHs and their controls) and the absorbance (Abs) was detected at λ = 730 nm between 12 s and 10 min, at 25 °C. The final concentration of all the antioxidant drugs was 13 µM. For an appropriate comparison, the ABTS assay was also tested on the free drugs: 2.9 mL of the diluted ABTS^•+^ solution in bi-distilled water were added by 0.1 mL of AX, RV or CU solutions in DMSO and the Abs was detected at λ = 730 nm. The final concentration of the free antioxidant drugs was the same as the loaded ones (13 µM). The antioxidant activity (AA) was calculated at 6 min, by using the following equation:

$$\%\;AA=\;\frac{Abs\;ABTS-Abs\;sample}{Abs\;ABTS}\times 100$$

#### 2.2.10. Cell Viability

MTS Test: 10,000 cells/well (175 µL of cell suspension in complete DMEM) were seeded in a 96-well plate and incubated for 48 h. HUVECs were then washed with 100 µL PBS, added by 175 µL of fresh complete DMEM and treated with 13 µL of PBS (healthy HUVECs) or 13 µL of 150 µM AM (oxidatively stressed HUVECs) for 1 h at 37 °C in the dark. Cells were washed with 100 µL PBS, added by 175 µL of fresh complete DMEM and incubated with 25 μL of samples (AX/NHs or NHs in PBS) at specific final concentrations (ranging from 31 to 500 μg mL^−1^, dilution factor (df) = 1:2) and incubated for 24 h. The highest concentration of AX in AX/NHs was 10 µg mL^−1^ (corresponding to 16.5 µM, df = 1:2). For an appropriate comparison, cells received 25 μL of PBS. The medium was then removed, cells were gently washed with 100 µL PBS and added by 100 μL of complete DMEM. 20 µL MTS were added to each well and the number of viable cells was measured by reading the Abs at 490 nm with the TECAN Infinite 200PRO plate reader (Mannedorf, Switzerland). Results were processed by using i-Control 1.10 software. Each experiment was performed on 8 wells (*n* = 3).

#### 2.2.11. Cell Morphology

10,000 cells/well (400 µL of cell suspension in complete DMEM) were seeded in a Lab-Tek and incubated for 48 h. HUVECs were then washed with 100 µL PBS, added by 175 µL of fresh complete DMEM and treated with 13 µL of PBS (healthy HUVECs) or 13 µL of 150 µM AM (oxidatively stressed HUVECs) for 1 h at 37 °C in the dark. Cells were washed with 100 µL PBS, added by 175 µL of fresh complete DMEM and incubated with 25 μL of samples (AX/NHs or NHs in PBS) at the final concentration of 250 μg mL^−1^ and incubated for 24 h. For an appropriate comparison, cells received 25 μL of PBS. HUVECs were washed with 400 µL PBS, fixed with 200 µL 4% PFA for 30 min at 4 °C, washed twice with 400 µL PBS and added by 200 µL of 0.1% Triton for 5 min at 25 °C. Cells were washed again twice with 400 µL PBS and then treated with 200 µL DAPI (dilution 1:1000) and phalloidin (dilution 1:200) solution for 30 min at 25 °C. Cells were finally washed twice with PBS (400 µL), slides were fixed and then digital images were obtained and analysed using Nanozoomer digital pathology software (Hamamatsu, Japan).

#### 2.2.12. Cellular Antioxidant Activity of AX/NHs

Cellular antioxidant activity (CAA) test: 10,000 cells/well (175 µL of cell suspension in complete DMEM) were seeded in 96 well plates and incubated for 48 h. HUVECs were then washed with 100 µL PBS, added by 175 µL of fresh complete DMEM and treated with 25 μL of samples (AX/NHs or NHs in PBS) at the final concentrations of 250 and 500 μg mL^−1^) and incubated for 6 h at 37 °C. The highest concentration of AX in AX/NHs was 10 µg mL^−1^, corresponding to 16.5 µM. As negative control, cells received 25 μL of PBS, whilst as positive control cells received 15 µL of 300 mM NAC (corresponding to a final concentration of 22 µM). The medium was then removed, cells were carefully washed with 100 µL PBS and added by 130 μL of 5 µM DHE dye and incubated for 30 min in the dark at 37 °C. Then 10 µL of 150 µM AM were added and HUVECs were incubated for 1 h at 37 °C in the dark. Cells were washed with PBS, added by 100 µL PBS and fluorescence was red with the TECAN Infinite 200PRO plate reader, using an excitation wavelength at 500 nm and an emission wavelength at 600 nm. Results were processed by using i-Control 1.10 software. Each experiment was performed on 4 wells (*n* = 3).

#### 2.2.13. Statistical Analysis

CAA of AX/NHs or NHs: viable cell counts were calculated using four biological replicate count data (each derived from three technical replicate data). All data are normalised to the negative control (untreated cells that received PBS) and are expressed as the mean value ± standard deviation. Statistical significance was determined using four biological replicate data (*n* = 3) with a Mann–Whitney test by using SPSS 20 Software. Values of *p* < 0.05 were considered significant. Asterisk denotes statistically significant differences (*p < 0.05).

Cell Viability: viable HUVECs (MTS test) were calculated using three biological replicates (each derived from 8 wells). All data are normalised to the negative control (untreated cells that received PBS) and are expressed as the mean value ± standard deviation. Statistical significance was determined using 8 wells (*n* = 3) with One-way ANOVA analysis in Prism (GraphPad 5.0 Software, Inc., La Jolla, CA, USA). Differences between the two groups were determined by a Turkey’s multiple comparison test. Asterisks denote statistically significant differences (*p < 0.05; **p < 0.01; ***p < 0.005).

## 3. Results

### 3.1. Cell Binding/Uptake Kinetics of NHs in Healthy or Oxidatively Stressed HUVECs

**Figure 1:**
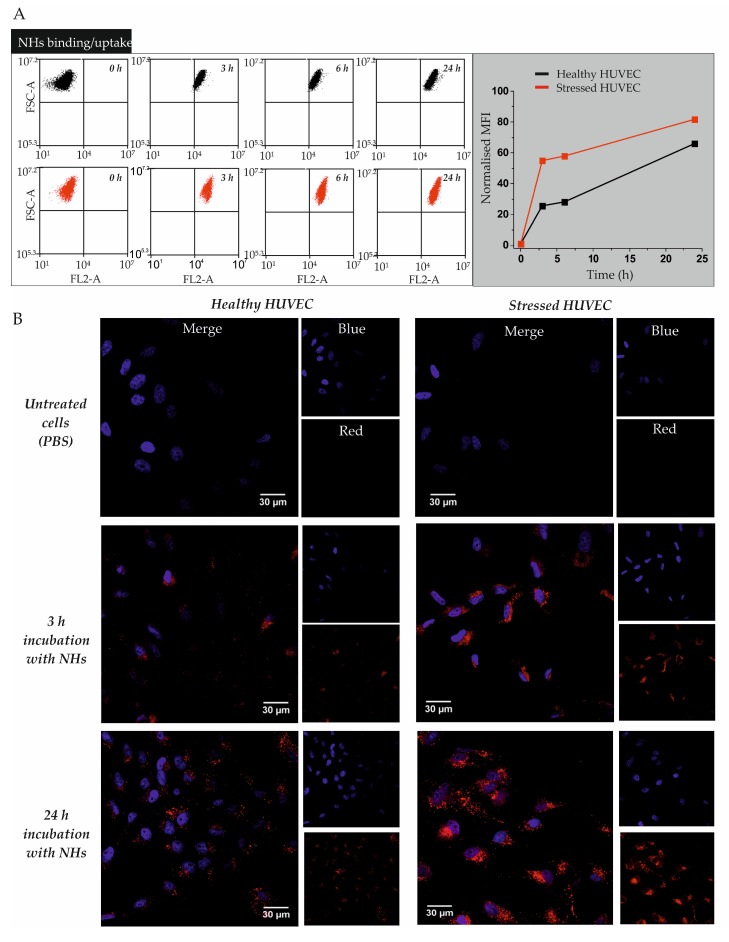
(**A**) Flow cytometry analysis of healthy or oxidatively stressed Human Umbilical Vein Endothelial Cells (HUVECs) incubated with rhodamine B isothiocyanate-nanohydrogels (Rhod-NHs). Cells were cultured in complete Dulbecco’s Modified Eagle Medium (DMEM) for 48 h and then incubated from 3 to 24 h with 100 μg mL^−1^ Rhod-NHs. (**B**) Confocal micro-graphs (scale bars: 30 μm) of healthy (left) or oxidatively stressed (right) HUVECs, incubated with 100 μg mL^−1^ Rhod-NHs for 3 or 24 h. Blue colour refers to nuclei incubated with 4′,6-diamidino-2-phenylindole (DAPI), whilst red colour refers to Rhod-NHs. Untreated cells received PBS.

Rhod dye was covalently linked to the hydroxyl groups of NHs [17] and the obtained fluorescent NHs were used for the binding/internalization kinetics study in healthy or oxidatively stressed HUVECs by flow cytometry (Figure 1A) and confocal microscopy (Figure 1B). Flow cytometry analysis evidenced that both, healthy and oxidatively stressed HUVECs, incubated for 3 h with Rhod-NHs, showed a significant increase in fluorescence intensity, indicating an evident binding/uptake of NHs within the cells, followed by a plateau up to 6 h; after 24 h the fluorescence intensity of HUVECs increased again (Figure 1A). Interestingly, the fluorescent intensity of oxidatively stressed HUVECs was almost two-fold higher than that of healthy HUVECs, at all the tested time points, suggesting that HUVECs are able to bind/take up more NHs in stress conditions. According to confocal microscopy analysis, NHs are located into vesicle-like structures in the perinuclear area, suggesting an intracellular location of NHs (Figure 1B). A similar outcome was already obtained, by incubating human keratinocytes (HaCaT) with Rhod-NHs [17]. Moreover, micro-graphs of oxidatively stressed cells showed a higher red fluorescence than that observed for healthy cells, confirming that HUVECs take up more NHs in stress conditions.

### 3.2. Preparation and Characterisation of AX/NHs, RV/NHs and CU/NHs Systems

**Figure 2:**
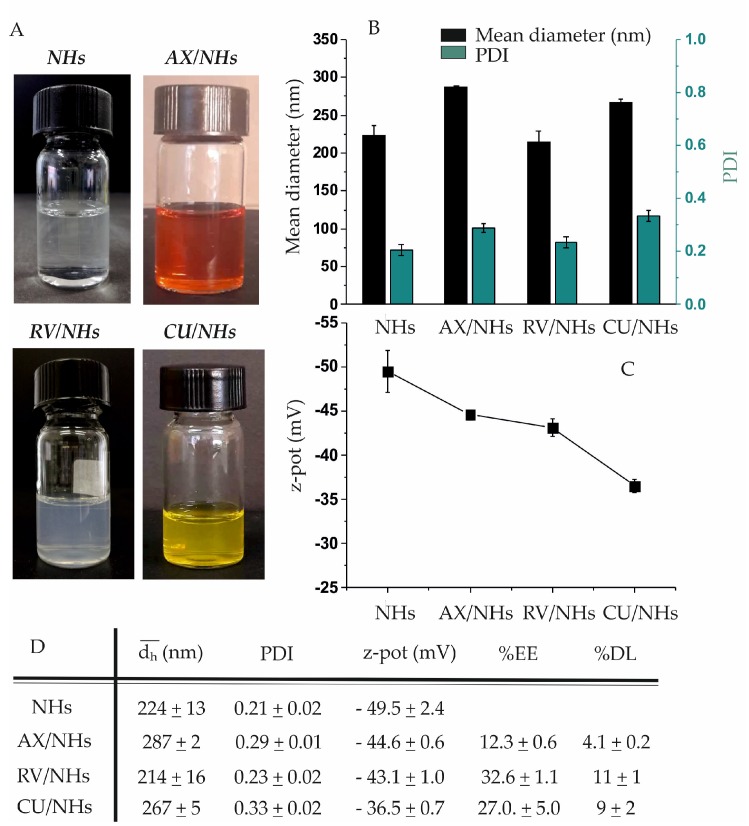
(**A**) Pictures of NHs, astaxanthin/nanohydrogels (AX/NHs), resveratrol/nanohydrogels (RV/NHs) and curcumin/nanohydrogels (CU/NHs) samples. (**B**) mean hydrodynamic diameter (d_h_) and polydispersity index (PDI) and (**C**) ζ-potential (ζ-pot) of free NHs, AX/NHs, RV/NHs and CU/NHs. Samples were characterised with dynamic light scattering (DLS) (**D**) Table summarising the physico-chemical properties (d_h_, PDI and ζ-pot), the encapsulation efficiency (EE%) and the drug loading (DL%) of AX/NHs, RV/NHs and CU/NHs.

AX, RV and CU, which are three natural antioxidant molecules poorly soluble in water, were loaded into NHs with the aim to enhance their solubilisation in aqueous environments, their intracellular accumulation as well as their therapeutic efficacy. NHs aqueous suspensions, obtained by autoclaving [28], were added by AX, RV or CU (according to the experimental procedures described in Section 2.2.5 and Section 2.2.6), for drug encapsulation. The obtained AX/NHs, RV/NHs and CU/NHs (Figure 2A) were purified from the free antioxidant drugs by centrifugation (4000 rpm for 10 or 20 min) and the EE% and the DL% were evaluated with HPLC (RV/NHs and CU/NHs) or with a UV-Vis spectrometer (AX/NHs). The amounts of entrapped drugs are reported in Figure 2D: RV loading (%EE, 32.6 ± 1.1) was similar to that of CU (%EE, 27.0 ± 5.0) and higher than that of AX (%EE, 12.3 ± 0.6). This may be due to the lower MW (smaller size) of RV or CU compared to AX. AX/NHs, RV/NHs and CU/NHs were characterised in terms of d_h_, PDI and ζ-pot, as summarised in Figure 2B–D, showing average sizes of 287 nm (AX/NHs), 214 nm (RV/NHs) and 267 nm (CU/NHs), which appear to be dependent on the MW of the drugs (AX > CU > RV). The ζ-pot net values of loaded-NHs (ranging from ≈ |45| to |37| mV) were lower than those of unloaded NHs (≈ |49| mV), but high enough to ensure a good stability of the nano-formulations.

### 3.3. Antioxidant Activity of AX/NHs, RV/NHs and CU/NHs Samples in Tube and in Vitro

**Figure 3:**
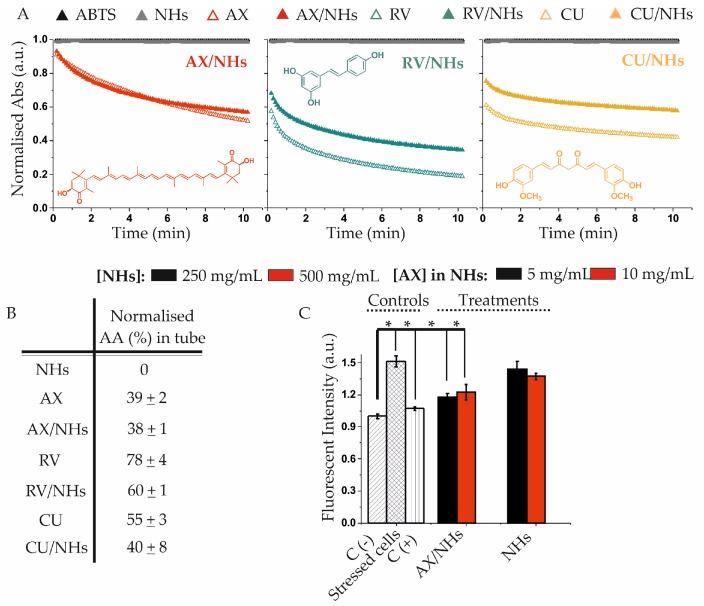
(**A**) Normalised Abs (λ = 730 nm) of AX/NHs, RV/NHs, CU/NHs and their controls versus 2,2′-azino-di-(3-ethylbenzthiazoline sulfonic acid) (ABTS). AX, RV and CU (loaded into NHs) were studied at the final concentration of 13 µM, respectively. Their absorbance (Abs) was compared to that of the free drugs at the same concentration. (**B**) Antioxidant activity (AA, %) of AX/NHs, RV/NHs, CU/NHs and their controls at 6 min. Data are expressed as the mean value ± standard deviation; experiments were performed in triplicate (*n* = 3). (**C**) Cell antioxidant activity (CAA) of AX/NHs and free NHs in oxidatively stressed HUVECs at two different concentrations (corresponding to NHs concentration of 500 and 250 µg mL^−1^ and AX concentration of 5 µg mL^−1^ (8 µM) and 10 µg mL^−1^ (16 µM). Experiments were performed on 4 wells (*n* = 3). Statistical significance was determined with Mann-Whitney test and asterisk denotes statistically significant differences (*p < 0.05).

Once loaded into NHs, the AA of AX, RV and CU were checked with the ABTS test [29] and compared to that of the free drugs. ABTS assay measures the ability of the antioxidant molecules to scavenge the ABTS^•^^+^, which is generated by the reaction of ABTS salt with a strong oxidising agent, such as potassium persulfate. The reduction of the blue-green ABTS^•^^+^ coloured solution (due to the presence of hydrogen-donating antioxidant molecules) is due to the suppression of its characteristic absorption spectrum at λ = 730 nm. Results evidenced that free NHs do not show any AA, whilst RV has the highest AA, followed by CU and finally by AX. The AA of AX molecules did not change when they were loaded into NHs (Figure 3A,B); conversely, loaded molecules of RV and CU lost 23 and 27% of AA, respectively, compared to those of the starting drugs. This result may be due to the longer encapsulation process (14 h for RV and CU and 5 h for AX), needed to obtain stable nano-formulations with suitable physico-chemical properties. The highest AA was evidenced by RV/NHs (60%), whilst AX/NHs and CU/NHs showed similar AA (38 and 40%, respectively). 

According to the obtained results, AX/NHs sample was chosen for the *in vitro* test. In HUVECs, ROS were generated with AM, which is a molecule able to inhibit succinate oxidase, NADH oxidase and the mitochondrial electron transport between cytochrome b and c [30]. The inhibition of electron transport causes both the collapse of the proton gradient across the mitochondrial inner membrane and the production of ROS. DHE dye was employed for monitoring the ROS production. Results show the OS occurs when HUVECs are incubated with AM (final concentration 11 µM), such ROS production is reduced by NAC, which is the antioxidant typically employed as a positive control. When HUVECs were incubated with AX/NHs, the nano-formulation showed a similar CAA as NAC, thus confirming the capability of AX/NHs to inhibit the ROS production in oxidatively stressed HUVECs, at all tested concentrations (Figure 3C). In contrast, unloaded NHs did not show any CAA in these conditions (Figure 3C).

### 3.4. Cell Viability and Morphology

**Figure 4:**
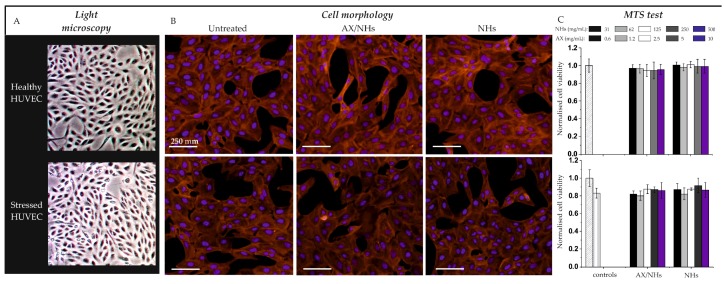
(**A**) Light microscopy micro-graphs of healthy or oxidatively stressed HUVECs. (**B**) Fluorescence microscopy micro-graphs (cell morphology) of healthy or oxidatively stressed HUVECs incubated with AX/NHs or free NHs at the concentration of 250 µg mL^−1^ (NHs) and 8 µM (AX) for 24 h. Nuclei were stained with DAPI (blue), whilst actin filaments were stained with phalloidin (red). (**C**) Cell metabolism (MTS test) of healthy or oxidatively stressed HUVECs. Cells were incubated with AX/NHs or free NHs over 24 h, at several concentrations (ranging from 31 to 500 µg mL^−1^ (NHs) and from 1 to 16 µM (AX)). Data were normalised to the negative control (HUVECs that received PBS). Statistical significance was determined with Mann-Whitney test and asterisk denote statistically significant differences (*p < 0.05). Statistical significance was determined with One-way ANOVA analysis and differences between the two groups were not detected. Results are expressed as the mean value ± standard deviation and experiments were performed on 8 wells (*n* = 3).

To determine the effects of AX/NHs and free NHs on cell viability, healthy or oxidatively stressed HUVECs (Figure 4A) were incubated with AX/NHs or free NHs over 24 h, at several concentrations (ranging from 31 to 500 µg mL^−1^ (NHs) and from 1 to 16 µM (AX)). Both cell morphology (Figure 4B) and MTS (Figure 4C) showed that nano-formulations were not toxic to healthy or oxidatively stressed HUVECs, as neither HUVECs morphology nor metabolism was significantly affected by any of the tested concentrations after 24 h (Figure 4B,C). However, stressed HUVECs appear to be slightly smaller and a bit less metabolically active (18%) than healthy HUVECs.

## 4. Discussion

In order to prepare NHs, the carboxyl groups of HA were covalently linked to the functionalised cholesterol (CH-Br), leading to the amphiphilic HA-CH polymer (Df = 15, mol%) [18]. HA-CH macromolecules are able to self-assemble into nano-sized structures (NHs) after a suitable treatment (i.e., autoclaving at 121 °C for 20 min) [28]. Such nanoparticles are formed by internal hydrophobic domains and by external hydrophilic layers.

A detailed characterisation of NHs systems, their dimensions and spherical shape, their soft nature and their swelling capability, was already reported in previous works [18,20].

Furthermore, the ability of NHs to entrap hydrophilic antibiotics (e.g., gentamicin and levofloxacin) and to enhance their antimicrobial activity against intracellular pathogens was evidenced in keratinocytes (HaCaT) [17] and HeLa cells [31].

In the present work, NHs were physically loaded with natural and poorly water-soluble antioxidant compounds. The NHs capability to accumulate into healthy or oxidatively stressed endothelial cells (HUVECs) and to entrap AX, RV and CU molecules, as well as to retain/enhance their antioxidant activity, both in tube and *in vitro*, was investigated. Flow cytometry and confocal microscopy data (Figure 1) showed that NHs enter HUVECs and accumulate in their perinuclear area; a similar result was obtained in keratinocytes and reported in a previous work [17]. Moreover, the experiments clearly show that NHs were taken up by oxidatively stressed HUVECs more than by the healthy ones (~ two-fold). Such results suggest NHs may deliver antioxidants inside the cells and preferentially to endothelial cells that are under oxidative stress. However, additional studies are necessary to elucidate the internalisation mechanism with which NHs bind/enter HUVECs. 

AX, RV and CU molecules were successfully loaded into NHs, leading to nano-formulations with suitable size, PDI and ζ-pot values (Figure 2) for biomedical applications. Furthermore, as expected, the apparent water solubility of AX, RV and CU was significantly enhanced when loaded into NHs, offering the opportunity to improve the administration, bioavailability and therapeutic efficacy of these compounds. The encapsulation time of RV and CU (14 h) was longer than that of AX (5 h), as RV/NHs and CU/NHs did not show suitable physico-chemical properties at shorter encapsulation time points. On the other hand, it was observed that the longer encapsulation time negatively affected the AA of the molecules. In fact, once loaded into NHs, the AA of AX (AX/NHs) was fully retained, whereas the AA of RV/NHs and CU/NHs decreased of 23 and 27%, respectively, when compared to the starting drugs (Figure 3A). Hence, the shortest encapsulation time was chosen for each nano-system, to obtain both suitable physico-chemical and antioxidant properties. Since RV and CU evidenced the loss of the AA after loading (possibly due to the partial oxidation of the molecules after the long encapsulation process), AX/NHs was selected as the best nano-system for the *in vitro* studies. In order to test the CAA of AX/NHs, HUVECs were oxidatively stressed with AM, a molecule able to enhance the ROS production at the mitochondrial level. The ROS increase can be studied by DHE, a compound commonly used for detecting cytosolic superoxide [32]. Upon reaction with the ROS species, DHE forms a red fluorescent product with maximum excitation and emission peaks at 500 and 580 nm, respectively. After incubation with AM, HUVECs showed a significant increase in fluorescence (~ 1.5-fold), thus confirming the oxidative stress (Figure 3B) occurred. The stressed HUVECs were incubated with AX/NHs or free NHs in order to investigate their CAA capability; NAC was tested as a positive control. Results show AX/NHs were able to inhibit the oxidative stress in HUVECs as much as NAC, at all the tested concentrations suggesting that I) NHs can effectively deliver AX into HUVECs; II) AX retains its antioxidant activity after encapsulation. Free NHs did not show any AA inside the cells. Promising results were also obtained by loading AX into cyclodextrins and lipid nanoparticles as reported by other researchers [9,33,34]. Cyclodextrins/AX complexes were found to be efficient in delivering AX from the PVA cardiac patches [35] as well as from the pullulan-dextran natural matrix, in an ischemia reperfusion rat model [33]. Both AX/NHs and free NHs did not evidence any significant toxicity against healthy or oxidatively stressed HUVECs, at all tested concentrations (Figure 4), as shown by cell metabolism data (MTS test) and morphology micro-graphs. 

These results suggest self-assembled HA-based NHs may represent a suitable candidate for the delivery of natural antioxidants. Furthermore, it is worth noting that HA is among the most widely used biopolymers in cosmetics. Hence, formulations based on antioxidants-loaded HA NHs may be developed and tested both in pharmaceutical and cosmetic fields [36].

## 5. Conclusions

HA-based NHs are capable to entrap natural and poorly water-soluble antioxidants, such as AX, RV and CU and to significantly enhance their apparent water solubility in aqueous media. The nano-formulations are able to neutralise ROS species, as evidenced by in tube and *in vitro* (AX/NHs only) studies and do not show any toxicity against HUVECs cells. Since previous results suggest NHs are highly taken up by oxidatively stressed HUVECs, they may represent a suitable nano-carrier for delivering antioxidant molecules intracellularly. *In vivo* studies will help to evaluate the potential use of these systems for the medical treatment of OS pathologies, particularly in cardiovascular diseases such as ischemia/reperfusion (I/R) injuries.
